# Some Biogenetic Considerations Regarding the Marine Natural Product (−)-Mucosin†

**DOI:** 10.3390/molecules24224147

**Published:** 2019-11-15

**Authors:** Jens M. J. Nolsøe, Marius Aursnes, Yngve H. Stenstrøm, Trond V. Hansen

**Affiliations:** 1 Faculty of Chemistry, Biotechnology and Food Science, Norwegian University of Life Sciences, P.O. Box 5003, 1433 Ås, Norway; yngve.stenstrom@nmbu.no (Y.H.S.); t.v.hansen@farmasi.uio.no (T.V.H.); 2 Department of Pharmaceutical Chemistry, University of Oslo, P.O. Box 1068, 0316 Oslo, Norway; marius.aursnes@farmasi.uio.no

**Keywords:** ene-reductases, Diels-Alderases, marine hydrindane natural product, arachidonic acid metabolite, eicosanoid motif, tentative PUFA biogenesis, prostaglandins, *trans*-fused carbocycle, 7,8-disubstituted bicyclo[4.3.0]non-3-ene, concerted vs. discrete pathways

## Abstract

Recently, the identity of the marine hydrindane natural product (−)-mucosin was revised to the *trans*-fused structure **6**, thereby providing a biogenetic puzzle that remains to be solved. We are now disseminating some of our insights with regard to the possible machinery delivering the established architecture. Aspects with regard to various modes of cyclization in terms of concerted versus stepwise processes are held up against the enzymatic apparatus known to be working on arachidonic acid (**8**). To provide a contrast to the tentative polyunsaturated fatty acid biogenesis, the structural pattern featured in (−)-mucosin (**6**) is compared to some marine hydrinane natural products of professed polyketide descent. Our appraisal points to a different origin and strengthens the hypothesis of a polyunsaturated fatty acids (PUFA) as the progenitor of (−)-mucosin (**6**).

## 1. Introduction

As primary metabolites, polyunsaturated fatty acids (PUFAs) show forth comparatively modest complexity [1]. However, when not integrated as constituents of the eukaryotic cell membrane or serving as a fuel repository, further enzymatic transformation can result in a plethora of structurally diverse natural products [2,3,4,5]. Particularly, the marine environment has articulated the complexity by providing an array of unique naturally occurring carbocycles [6,7]. Furthermore, a number of secondary PUFA metabolites, isolated from sea-dwelling eukaryotes, have been attributed with architectural motifs that resemble mammalian physiological modulators [6,7,8].

Over the last two decades, there has been a change of paradigm in the conception of how inflammation is brought to a halt [9,10,11]. No longer viewed as a passive process, but, rather like a finely tuned clockwork, the return to cellular homeostasis is driven by the active involvement of several specialized oxygenated PUFA products [4,12,13,14,15,16,17]. Traditionally, prostaglandins have been categorized as a class of proinflammatory lipid mediators [18,19,20,21,22,23,24,25,26,27,28]. Yet, over time, research has nuanced their physiological role to be context-dependent. Consequently, there are also reports that detail potent anti-inflammatory action [29,30,31,32]. Considering this ambimodal behaviour, related structures might clarify interactions on a receptor level and advance understanding of pathophysiological aspects linked to neuroinflammation [33,34,35,36,37].

**Figure 1:**
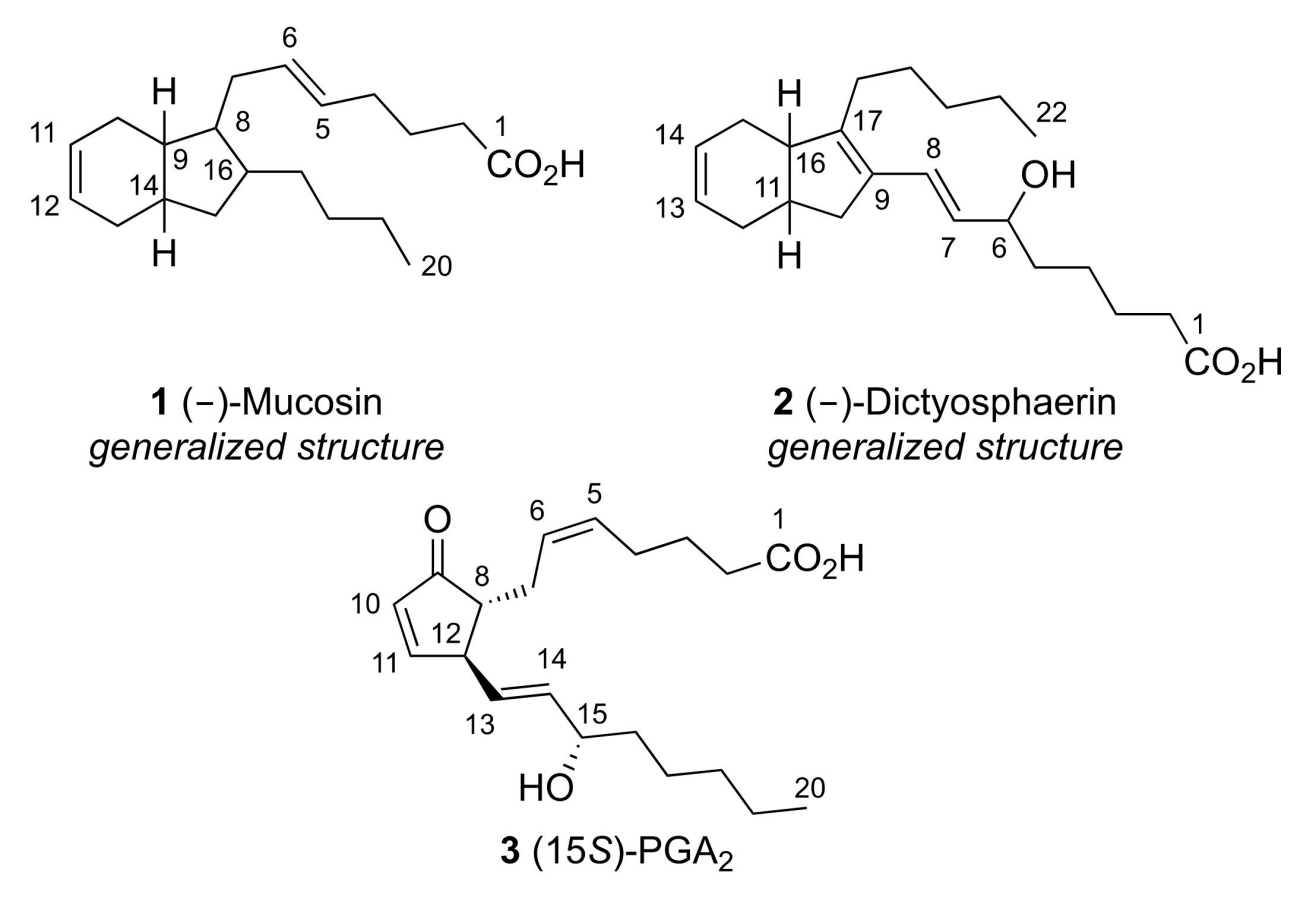
Generalized structures of mucosin and dictyosphaerin with a marine prostaglandin.

Amongst the profusion of structures brought to light through prospection of various marine habitats [38,39,40,41,42,43,44,45], the prostaglandin motif has been gleaned on several occasions [6,7,8]. Often, these marine carbocyclic oxylipins can be related directly to components found in the human inflammatory metabolome [46]. However, in some cases, the kinship to prostaglandins is more convoluted, as exemplified by the generalized structures of (−)-mucosin (**1**) and (−)-dictyosphaerin (**2**) (Figure 1) [47,48].

**Figure 2:**
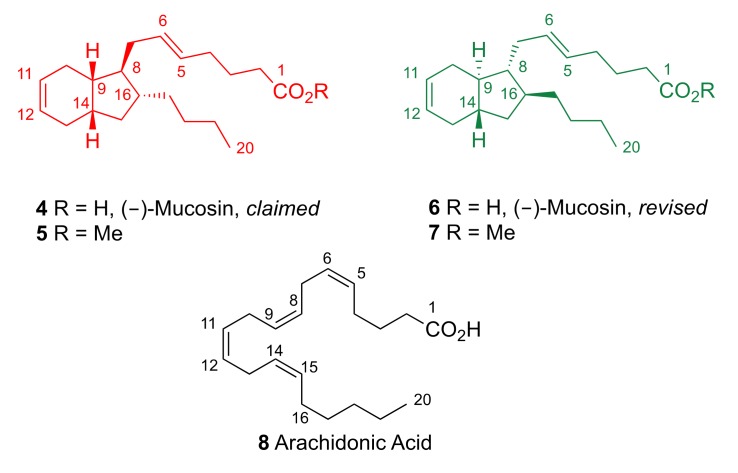
Claimed and revised structures of (−)-mucosin and relationship to arachidonic acid.

The pertinent architecture of these two marine natural products is entrenched in a polycyclic ring system. Thus, amounting to densely functionalized cyclopentanes, the cited compounds pose a challenge inasmuch as the relative stereochemistry is usually assigned on the basis of NMR alone. A fascinating development in natural product synthesis is the application of Hidden Markov Models (HMM) to aid structural elucidation of polyketide motifs [49]. Still, irrespective of how the analogy is reached, the fundamental aspects related above make these natural products interesting investigative targets. The authors of the present paper have been engaged in a successful campaign that ultimately established the correct structure of the marine eicosanoid (−)-mucosin (**1**) by stereocontrolled total synthesis [50,51,52]. In contrast to the claimed *cis*-fused structure **4**, it was clearly demonstrated that the natural product has the *trans*-fused structure **6** (Figure 2). Based on the revision, we herein discuss the implications of the fusion geometry and the overall topology in relation to the putative biosynthesis from arachidonic acid (AA) (**8**). To offer a juxtaposition, an earlier biogenetic proposition for certain marine hydrindane natural products is pitched against structure **6** with a polyketide origin in mind.

## 2. Results and Discussion

Isolated in 1997 from the mediterranean sea sponge *Renierea mucosa*, the original assignment of (−)-**1** was performed by Casapullo et al. on its methyl ester derivative [47]. Thus, subsequent to HRMS and IR, application of various NMR techniques established that the parent C_20_-compound encompassed a prostaglandin-like pattern superimposed on a bicyclo[4.3.0]non-3-ene scaffold. However, while arriving at the correct overall structure, the alleged and somewhat surprising *cis*-fused topology advocated for (−)-mucosin (**4**) was since proven wrong by us [50].

In order to unearth the true nature of the publicized sponge metabolite, we performed an asymmetric total synthesis of the most likely candidate **6** [52]. This nomination was guided by a biogenetic conjecture and was further supported by DFT calculations on selected stereopermutants. Upon evaluation of the relative energies obtained in silico from the geometry-optimized structures, it was clear that the absence of A^1,2^ strain between H8, H9, H14 and H16 was a decisive factor and consequently the *trans*-fused topology present in **6** was singled out. Finally, the synthetic material **7** from the enforced strategy was matched against the data given for the natural product methyl ester derivative **5**. This clenched the argument and pinpointed (−)-mucosin (**6**) as the compound isolated from *R. mucosa* [47].

The metabolic pathway giving rise to (−)-mucosin (**6**) has currently not been made the subject of any investigation. Though, when disclosing the isolation and simultaneously broadcasting their structural assignment, Casapullo et al. suggested AA (**8**) as a possible origin for the marine natural product [47]. Considering the general lay out of the bicyclic scaffold and the source organism, this seems to be a plausible assumption. What is more, it provides a link to the prostaglandin class and other secondary metabolites of AA (**8**), as well as an enzymatic apparatus that has been studied in detail [6,7,53]. On the other hand, despite the endorsing traits, the professed enzymatic apparatus has not left a legible tell-tale sign that heralds its action in the biosynthesis of the bicyclo[4.3.0]non-3-ene ring system. It is, therefore, impossible to dismiss a polyketide pathway that bypasses AA (**8**) in the assembly of (−)-mucosin (**6**) [54].

In keeping with the above preamble, we will present some alternative hypothesis. Thus, when considering biogenesis, irrespective of the pathway taken, some fundamental requirements must be fulfilled in terms of the organization of a cyclic transition state leading to the *trans* fused skeleton. First, upon establishing a chiral foothold, the asymmetric information must unequivocally be transmitted to the seat of reaction. Secondly, as the nascent ring system is subject to transannular strain between the C8 and C14 olefins due to eclipsing interactions, the organization of the reacting acyclic precursor must necessary reflect a minimized conformation. Thirdly, if some pericyclic mechanism is invoked, the reaction must be symmetry allowed in the ground state and that the resulting geometry follows as a consequence.

### 2.1. Pericyclic Pathway Involving AA (**8**)

If accepting AA (**8**) as the progenitor of (−)-mucosin (**6**), the inductor of asymmetry might be searched out amongst the enzymes active on the PUFA-backbone. More explicitly, arachidonate 5-lipoxygenase (5-LOX) stereoselectively converts AA (**8**) to the corresponding 5-hydroperoxide **9** in both terrestrial and marine organisms [53,55]. Furthermore, two antipodal isoforms, 5*S*- and 5*R*-LOX, have been identified [56,57]. Thus, assuming that the biosynthetic pathway leading to (−)-mucosin (**6**) is initiated by conversion of AA (**8**) to the 5*R*-hydroperoxide **9** by 5*R*-LOX, several ensuing scenarios can be envisioned.

**Scheme 1:**
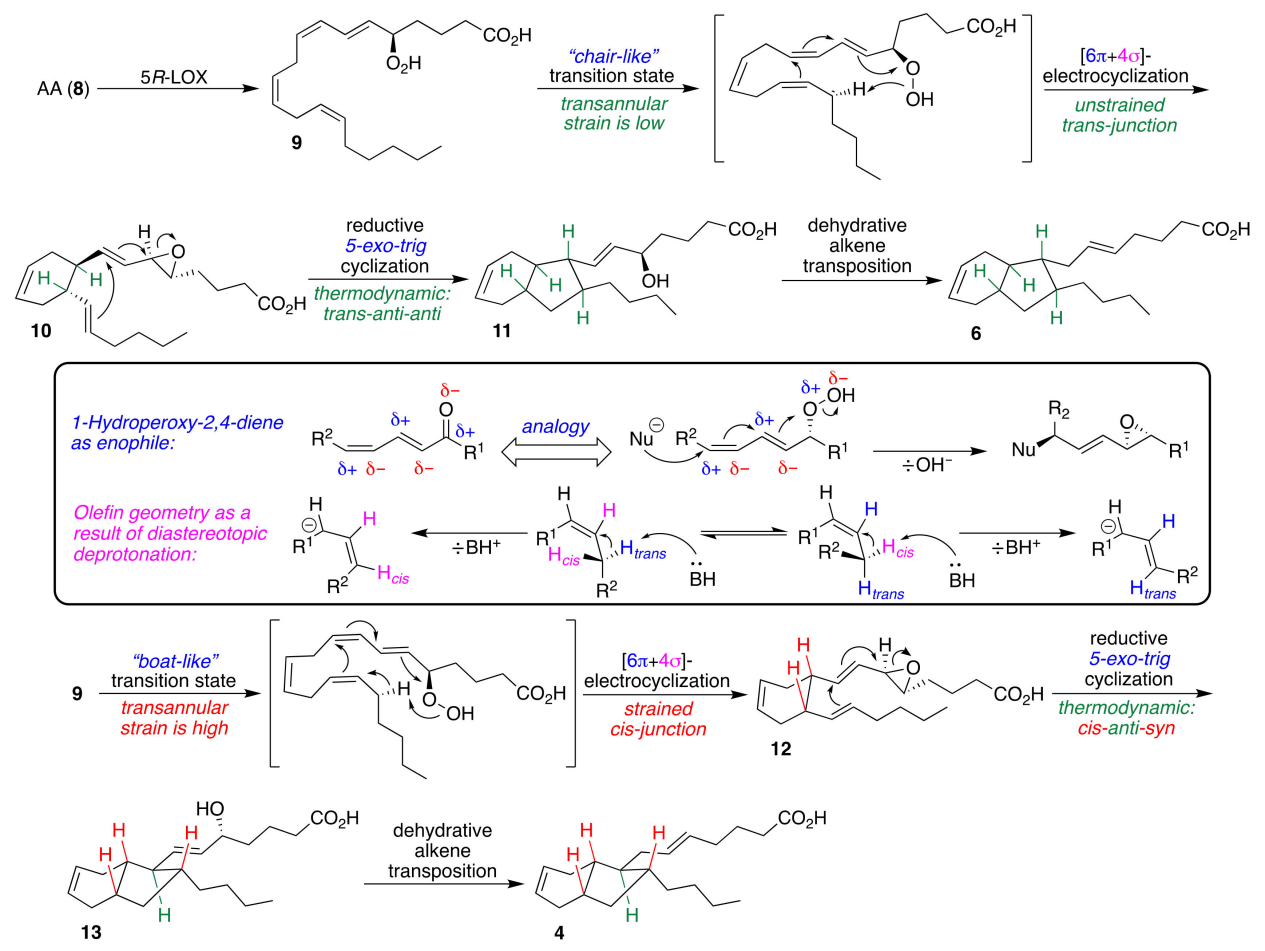
Pericyclic pathway of AA (**8**) catalysed by 5*R*-LOX, favouring structure **6** over structure **3** via equilibrating macrocyclic TSs and thermodynamic control governed by strain interactions.

The recent years have seen an increased focus on the ene reaction in terms of biological relevance [58]. Indeed, for the formation of 5*R*-hydroperoxide **9** itself, one might cite an ene-type reaction between molecular oxygen and the allylic motif spanning C5–C7 in **6**. Generally, whether or not the process follows a concerted or a stepwise mechanism is a different matter; the observed outcome only allows a circumstantial chain of evidence. However, it provides a concrete backdrop on which to rationalize the transfiguration of a proposed substrate into the natural product under scrutiny. Thus, with this proviso in mind, the polar ene-type reaction seems particularly interesting as a motive force in the biogenesis of (−)-mucosin (**6**). The key argument draws on an analogy between the 5*R*-hydroperoxy-6*E,8Z*-diene present in **9** and a vinyl ketone (Scheme 1). By invoking a polar acceptor moiety, the HOMO–LUMO gap is decreased relative to the formal ene reaction, thereby greatly enhancing the orbital overlap between the reacting termini [59,60,61]. Additionally, by adopting a macrocyclic transition state that allows the 5*R*-hydroperoxy group to intercede with one of the diastereotopic C16 methylene protons, a holdfast is formed and a continuous array of overlapping orbitals may be attained. In this manner, the stereochemistry set at the behest of 5*R*-LOX simultaneously dictates the topology at the points of fusion (C9 and C14) and the olefin geometries (C7 and C15). Formally, this is a [6π + 4σ]-electron system, making the concerted reaction a thermally allowed, disrotatory, suprafacial, process. Yet, prior to annealing the six-membered ring, it is in fact the sampling of the conformational space available to **9** that reigns supreme. Two energetically discernible transition states may be described for **9**, wherein the ephemeral macrocycle either adopts a “chair”-like or a “boat”-like conformation. Upon further inspection, transannular strain is minimized in the “chair”-like conformation and progress along this reaction coordinate leads to the *trans*-disubstituted cyclohexene **10** free of A^1,2^ strain. By contrast, the “boat”-like conformation will result in A^1,2^ strain as the *cis*-disubstituted cyclohexene **13** is formed. Consequently, the cumulative weight of enzymatic induction, conformational bias and stereoelectronic effects converge on one geometry and one topology, namely that which is found in *trans*-fused **6**. As a direct consequence of the suggested polar ene-type reaction, an unambiguous functional pattern is established, which sets the stage for a subsequent Michael-type reductive cyclization to render the complete *trans*-bicyclo[4.3.0]non-3-ene skeleton. Thus, propped up by the cyclohexene ring in **10**, the *trans*-vinyl epoxide group attached to C9 acts as the electrophile, while, depending on which of the diastereotopic protons at C16 that was abstracted, a *cis*- or a *trans*-vinyl group attached to C14 acts as the nucleophile. Governed by thermodynamics, and elicited by a reductant such as NADPH or a tyrosin residue, a *5-exo-trig* cyclization is then postulated to forge the cyclopentane ring with an *anti*-configuration for the two substituents (C9 and C16). Eventually, the overall topology of the four chiral carbons featured in the ring system is a propagation of the stereocentre established by 5*R*-LOX and the anticipated outcome falls within the reasoning of the Curtin–Hammett principle [62,63]. Finally, after the stereocentre at C5 has served its turn, it is obliterated in a dehydrative alkene transposition. Consequently, 5*R*-LOX furnishes (−)-mucosin (**6**) in a traceless manner.

### 2.2. Cationic Pathway Involving AA (**8**)

**Scheme 2:**
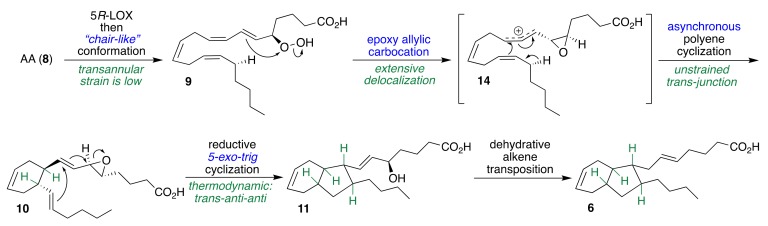
Asynchronous carbocationic pathway of AA (**8**) catalysed by 5*R*-LOX leading to structure **6** via an equilibrating macrocyclic TS and thermodynamic control governed by strain interactions.

As an alternative to the proposed pathway, it is possible to envision the cyclization of **9** taking place via a formally charged species (Scheme 2). Here, our underlying reasoning piggybacks on a conceptual proposal by Gerwick, wherein **9** is transformed to an epoxy allylic carbocation **14** [64]. However, this modification has some implications on the stereochemical arguments, as it no longer constitutes a pericyclic process. Although the transformation involves a [4π + 2σ]-electron system, there is no closed loop of continuously overlapping orbitals and the reaction proceeds in a stepwise manner. Yet, drawing on the same conformational arguments as above (vide supra), the reaction will result in the *trans*-disubstituted cyclohexene **10**, and consequently, there is a convergence of pathways, irrespective of whether it is concerted or discrete. A noteworthy aspect of the detailed proposition, is that the cyclization of epoxy allylic carbocation **14** intercepts the step leading to antipodal leukotriene A_4_ (*ent*-LTA_4_) (**15**).

### 2.3. Anionic Pathway Involving AA (**8**) via ent-LTA_4_ (ent-**15**)

**Scheme 3:**
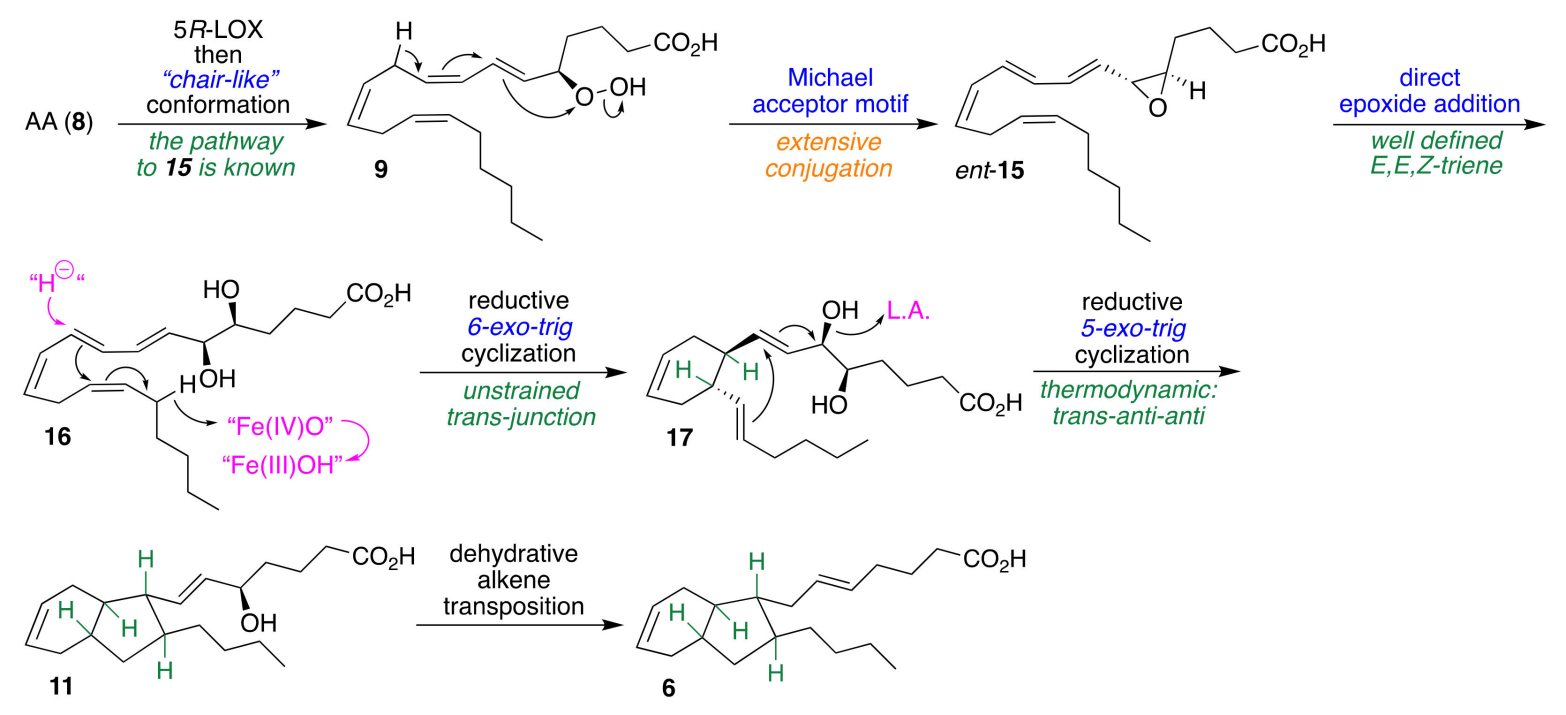
Quasi-synchronous carbanionic pathway of AA (**8**) catalysed by 5*R*-LOX and a formal ene-reductase leading to structure **6** via equilibrating macrocyclic TS and thermodynamic control governed by strain interactions.

*ent*-LTA_4_ (*ent-***15**) may itself be postulated to be a substrate for the biosynthesis of (−)-mucosin (**6**) (Scheme 3). The prominent 5,6-epoxy-7*E*,9*E*,11*Z*-triene moiety is a labile motif and susceptible to direct nucleophilic attack on the epoxide, as well as conjugate addition across the triene system [65,66]. Yet, the reactive manifold is closely controlled on a cellular level. Thus, while the half-life of free LTA_4_ (**15**) has been determined to be between 3–10 s [67,68,69], the association to albumin or a fatty acid binding protein (FABP) prolongs the duration well beyond the minute range [67,70]. In a physiological context, this differentiation is a key as LTA_4_ (**15**) is the central compound in an enzymatic pathway leading to the leukotriene class of inflammatory mediators [53,71,72]. Therefore, in terms of our deliberations on the biosynthesis of (−)-mucosin (**6**), the third alternative is focused on a metabolite of *ent*-LTA_4_ (*ent-***15**), which is named (5*R*,6*R*)-diHETE (**16**). Noticeably, it is a dihydroxylated PUFA that is endorsed by reports detailing isolation from the marine environment [73]. Furthermore, non-enzymatic hydrolysis of LTA_4_ (**15**) also yields (5*R*,6*R*)-diHETE (**16**) [74]. Thus, up to this point, everything is in accordance with a known enzymatic downstream cascade [53], starting with the action of 5*R*-LOX on AA (**8**). However, we are now entering into uncharted territory, as the next step is a reductive *exo*-cyclization of **16**, requiring the concerted action across the double bonds at C9 and C14. Formally, the resulting *trans*-disubstituted cyclohexene **17** is then a product of an ene-reductase [75,76], where a hydride is compelled to react across an activated system that properly aligns the reacting termini. As, previously argued, the presence of a chiral foothold creates a conformational bias that restricts the reactive assembly. Generally, the ene-reductases, depending on whether the process takes place in eukaryotes or prokaryotes, utilize NADPH or FMNH2 to reduce electron-deficient alkenes, coaxed along by some simultaneous Lewis acidic coordination to a polar function, such as an ester, ketone or aldehyde. Synthetically, it has been demonstrated that the ene-reductases are competent at forming C–C bonds [77]. However, a brief glance at **16** reveals that the reacting termini cannot be activated in a similar manner as above. Instead, it is suggested that the allylic position at C16 is activated by a heme-containing enzyme [78], such as CYP P450 or an oxygenase, while NADPH concomitantly instigates a cyclization by adding a hydride to C10. By delivering **17** as the product, the compound is at the same oxidation state as **10** in the two earlier propositions. Thus, by a Lewis acidic interaction, **10** and **17** converge on the same end point to furnish (−)-mucosin (**6**).

### 2.4. Polyketide Pathways

**Scheme 4:**
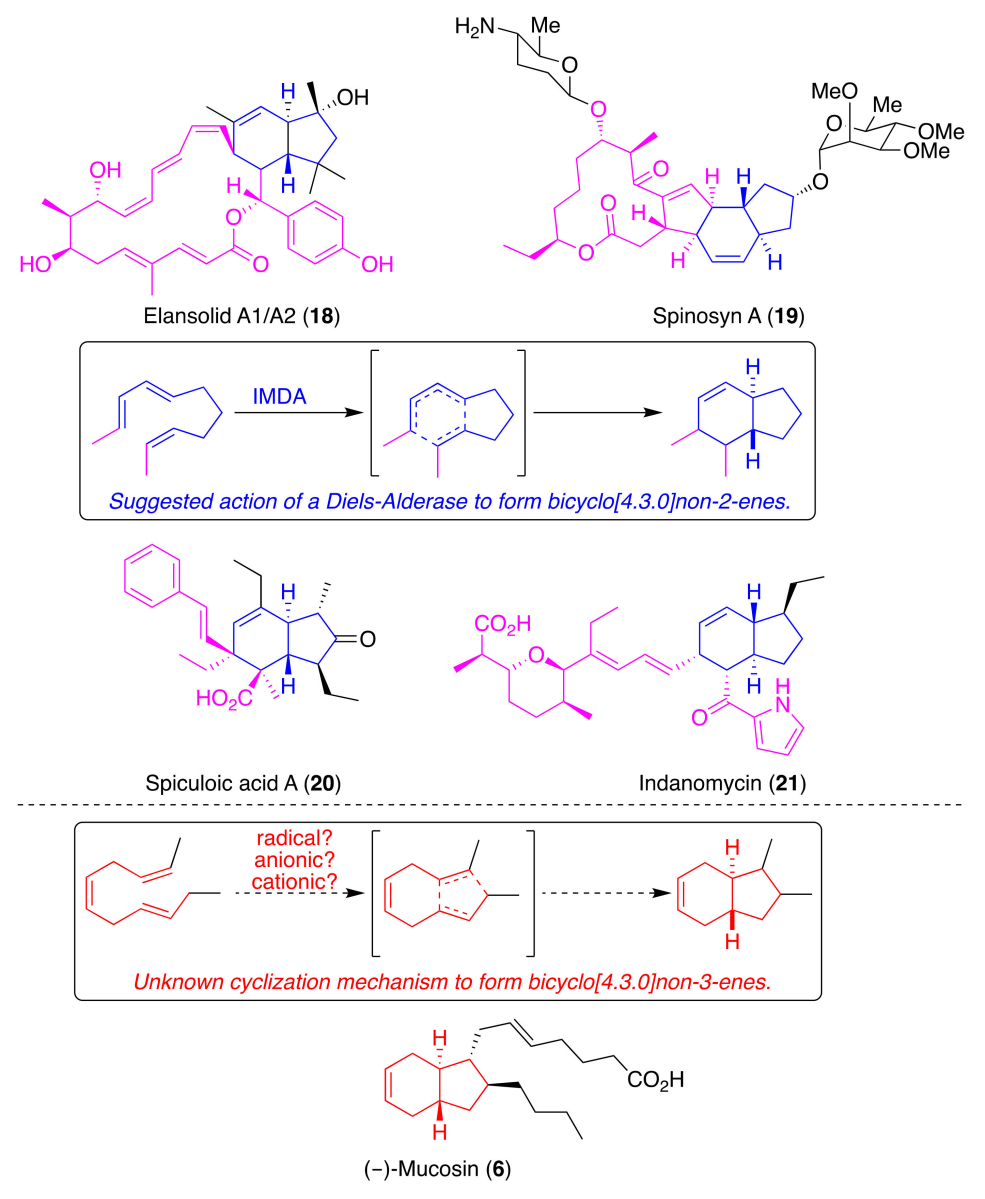
Contrasting the substitution pattern of marine hydrindane natural products embracing the bicyclo[4.3.0]non-2-ene motif with **6** and the implications for a biogenetic discussion.

A polyketide pathway cannot be dismissed to have superseded AA (**8**) in the biosynthesis of (−)-mucosin (**6**). In this sense, the fatty acid synthase (FAS) becomes synonymous with a polyketide synthase (PKS) set to shape the fused carbocycle through the modular machinery of malonyl CoA [79]. There are, in fact, a number of marine natural products with a hydrindane motif that have been ascribed to a polyketide origin. Of particular significance are spinosyn A (**18**), elansolid A1/A2 (**19**), spiculoic acid A (**20**) and indanomycin (**21**), which have a *trans*-fused bicyclo[4.3.0]non-2-ene core as their common feature (Scheme 4) [80,81,82,83]. At the moment, these compounds are at the epicentre of an ongoing debate revolving around whether or not Diels-Alderases are a part of Nature’s enzymatic machinery [84]. As a retron, the fusion geometry and the position of the double bond within the bicyclo[4.3.0]non-2-ene architecture are the tell-tale signs of an intramolecular Diels-Alder reaction (IMDA) [85].

However, while (−)-mucosin (**6**) is indeed also a marine hydrindane natural product with a *trans* junction, the resemblance to compounds **18**–**21** is superficial. A closer look at the layout reveals that the *trans*-fused bicyclo[4.3.0]non-3-ene core cannot be traced back to an IMDA retron without invoking a double bond isomerization in conjunction with cycloaddition. While this is not unlikely, the contrasting pattern of substitution is far more problematic. For compounds **18**–**21**, the basic motif is a 4,5-disubstituted *trans*-bicyclo[4.3.0]non-2-ene, while (−)-mucosin (**6**) is a 7,8-disubstituted *trans*-bicyclo[4.3.0]non-3-ene. Thus, the mode of annulation for a hypothetical common polyketide origin differs fundamentally between the two hydrindane scaffolds and they cannot have arisen via the same apparatus. The mechanism involving cyclization of a polyketide backbone to furnish the entire bicyclic structure of (−)-mucosin (**6**) remains obscure. Activation of a position adjacent to the significant double bond is illustrated in the suggested pathways leading from AA (**8**). A polyketide pathway may also include some surrogate functionality that can interact with Lewis acidic centres and/or electronically modulate the double bond. Yet, given the abundance of AA (**8**) in the marine environment and the documented diversity of the derived carbocyclic products [6,7], it seems more plausible that the enzymatic machinery furnishing (−)-mucosin (**6**) is to be found out amongst those working on the PUFA family.

## 3. Conclusions

In some respects, the molecular make-up of (−)-mucosin (**6**) is indeed reminiscent of prostaglandins and the entailed biogenetic hypothesis links the two motifs together with AA (**8**) as a common denominator. As prostaglandins are also found in similar marine fauna, the enzymatic apparatus needed to convert AA (**8**) enantioselectively to the stipulated intermediates is well established [53,55,56,57]. However, it seems unlikely that the complete carbocyclic skeleton is formed in a single transformation, but rather in a process where a series of distinct intermediates fall under the sway of thermodynamics. In this sense, the *trans*-disubstituted cyclohexenes **10** and **17** have a closer kinship to the structural motif found in prostaglandins than (−)-mucosin (**6**) itself, as the enzymatic imprint is eventually obliterated at the end of a supposed biogenetic sequence. Thus, *trans*-disubstituted cyclohexenes **10** and **17** are interesting synthetic targets, since they are potential metabolites that can be tested in terms of their competency to form the fused ring-system. From a structural perspective, it is concluded that (−)-mucosin (**6**) does not share its biogenetic origin with polyketide marine natural products containing the *trans*-bicyclo[4.3.0]non-2-ene core.
